# Effectiveness and cost effectiveness of cognitive behavioral therapy (CBT) in clinically depressed adolescents: individual CBT versus treatment as usual (TAU)

**DOI:** 10.1186/1471-244X-13-314

**Published:** 2013-11-21

**Authors:** Yvonne Stikkelbroek, Denise HM Bodden, Maja Deković, Anneloes L van Baar

**Affiliations:** 1 Department of Child and Adolescent Studies, Utrecht University, PO Box 80.140, NL-3508 TC Utrecht, Netherlands

**Keywords:** Effectiveness, Cost effectiveness, Randomised controlled trial (RCT), Depression, Major depressive disorder, Cognitive behavioral therapy, Adolescents, Treatment as usual

## Abstract

**Background:**

Depressive disorders occur in 2 to 5% of the adolescents and are associated with a high burden of disease, a high risk of recurrence and a heightened risk for development of other problems, like suicide attempts. The effectiveness of cognitive behaviour therapy (CBT), cost-effectiveness of this treatment and the costs of illness of clinical depression in adolescents are still unclear. Although several Randomized Controlled Trials (RCT) have been conducted to establish the efficacy of CBT, the effectiveness has not been established yet. Aim of this study is to conduct a RCT to test the effectiveness of CBT and to establish the cost-effectiveness of CBT under rigorous conditions within routine care provided by professionals already working in mental health institutions.

**Method/Design:**

CBT is investigated with a multi-site, RCT using block randomisation. The targeted population is 140 clinically referred depressed adolescents aged 12 to 21 years old. Adolescents are randomly assigned to the experimental (*N* = 70, CBT) or control condition (*N* = 70, TAU). Four assessments (pre, post, follow up at 6 and 12 months) and two mediator assessments during treatment are conducted. Primary outcome measure is depression diagnosis based on a semi-structured interview namely the K-SADS-PL. Secondary outcome measures include depressive symptoms, severity and improvement of the depression, global functioning, quality of life, suicide risk, comorbidity, alcohol and drug use, parental depression and psychopathology, parenting and conflicts. Costs and treatment characteristics will also be assessed. Furthermore, moderator and mediator analyses will be conducted.

**Discussion:**

This trial will be the first to compare CBT with TAU under rigorous conditions within routine care and with a complex sample. Furthermore, cost-effectiveness of treatment and cost-of-illness of clinical depression are established which will provide new insights on depression as a disorder and its treatment.

**Trial registration:**

Dutch Trial register number NTR2676. The study was financially supported by a grant from ZonMw, the Netherlands organization for health research and development, grant number 157004005.

## Background

Depressive disorders in adolescents are among the most prevalent disorders with a high burden of disease [1] and high risk of recurrence [2,3]. Before entering adulthood, 14 to 25% of the adolescents have experienced at least one episode of a depressive disorder [2]. Besides the high prevalence [4-7], comorbid psychiatric diagnoses are often present [8-10]. In addition, a heightened risk exists for development of social problems, juridical problems, learning problems, substance abuse, negative life events, physical problems, teen pregnancies and suicide [2,9,11]. Therefore, it is important that depression is treated in an early stage with an effective treatment [2,12].

In the international literature, there is no consensus on the degree of effectiveness of psychotherapeutic interventions in depressed adolescents. In a meta-analysis, which included studies with a large diversity of investigated interventions, only a modest effect size of 0.34 was found [13]. Other meta-analyses calculated medium (0.72) [14] to large (1.27) effect sizes [15]. In a meta-analysis solely directed at Cognitive Behavioral Therapy (CBT) a medium effect size of 0.53 was found [16]. Although the effect size for CBT is promising, it also reflects the need for improved treatment of depression, as a large group of depressed adolescents will not recover after CBT treatment.

In addition, it is often discussed that effectiveness studies in depressed adolescents lack generalizability for clinical practice, because the study samples do not match the complex and severe cases in routine mental health care [13]. In this study, this issue is addressed by comparing CBT to treatment as usual within a referred clinically depressed group of adolescents.

Within this study an individual revision of the group CBT program “Coping with Depression course for Adolescents” (CWD-A) [17] will be investigated. Group CBT was adapted into an individual CBT format because it is much better applicable within mental health care than group CBT, for instance children can start treatment immediately. Several RCT’s were conducted with an American population, but not with clinically referred adolescents. Results repeatedly have shown that the CWD-A was more effective than control conditions [18,19] and treatment as usual [20,21]. As only one research group investigated the CWD-A, it is regarded as probably efficacious [22].

The costs of depression in adolescents have not been studied before. Knowledge about costs of depression is essential to motivate an increase in budgets for treatments. A recent cost-of-illness study on children with anxiety disorders shows that both the costs of school absence, as well as productivity loss of the parents are substantial [23]. Given the high degree of comorbidity of anxiety and depression and the fact that both disorders are internalising disorders, the same high costs are expected in adolescents with depression. Lynch and colleagues [24] investigated cost-effectiveness of a group based prevention course “Coping with Stress” in adolescents with a subclinical depression. It was concluded that group CBT was more cost effective in comparison to treatment as usual. However, intervention related costs like productivity costs, expressed as school absence, were not taken into account. Including these costs could indicate that cost-effectiveness is even higher.

Within intervention research in depressed adolescents, little is known about possible moderators and mediators of treatment. A lot of authors mention the necessity to investigate factors that may be involved as such [13,22].

In conclusion, the efficacy of CBT for the treatment of clinically depressed adolescents is established, but the effectiveness is not yet clear. Cost effectiveness of CBT in comparison to Treatment As Usual (TAU) and potentially important moderators and mediators have not been investigated yet. Effectiveness, cost effectiveness, and information on moderators and mediators are essential for the enhancement of the treatment for depression in adolescence in order to reduce the burden of adolescent depression and recurrence.

## Methods and design

### 

#### Aim of the study

**Figure 1:**
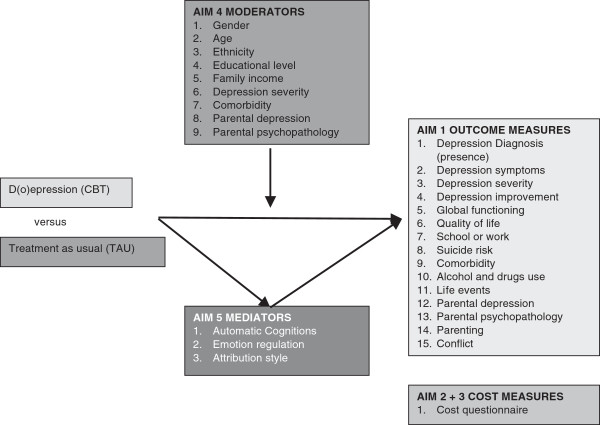
Aims, conditions, potential moderators and mediators evaluated in this study.

The aim of this study is to investigate the effectiveness and the cost-effectiveness of the individual CBT program the “D(o)epression course” in a sample of referred adolescents with a Depressive Disorder according to DSM-IV-TR [25] in a randomized controlled trial. We expect that CBT will be more effective than TAU (without CBT). Furthermore, cost-effectiveness of CBT and the cost of illness of clinical depression in adolescents will be established. Potential moderators (comorbidity, severity of depression, age, ethnicity, gender, suicidal thoughts and psychopathology in parents) and mediators (negative automatic thoughts, cognitive emotion regulation and attribution style) for the effectiveness of CBT will be studied. The role of non-specific treatment variables (therapeutic alliance, client expectancy, client satisfaction, treatment adherence) will be taken into account as well (see Figure 1).

#### Design

This study is designed as a multi-site, randomized controlled clinical trial in which individual CBT will be compared to TAU. Four multiple informant (adolescent, parent, therapist) assessments are done: prior to treatment (pre-test assessment), immediately after treatment (post-test assessment or after 15 sessions), 6 months after treatment (6 month follow-up) and 1 year after treatment (1 year follow-up). To investigate potential mediators two temporary assessments are conducted, each after 5 therapy sessions.

All sessions will take place within routine outpatient care within second order public mental health clinics to enhance external validity. All treatments in both conditions are delivered by psychologists with at least a master's degree-level and two years of experience within professional mental health care. These professionals are trained before delivering the treatments to enhance treatment integrity.

**Figure 2:**
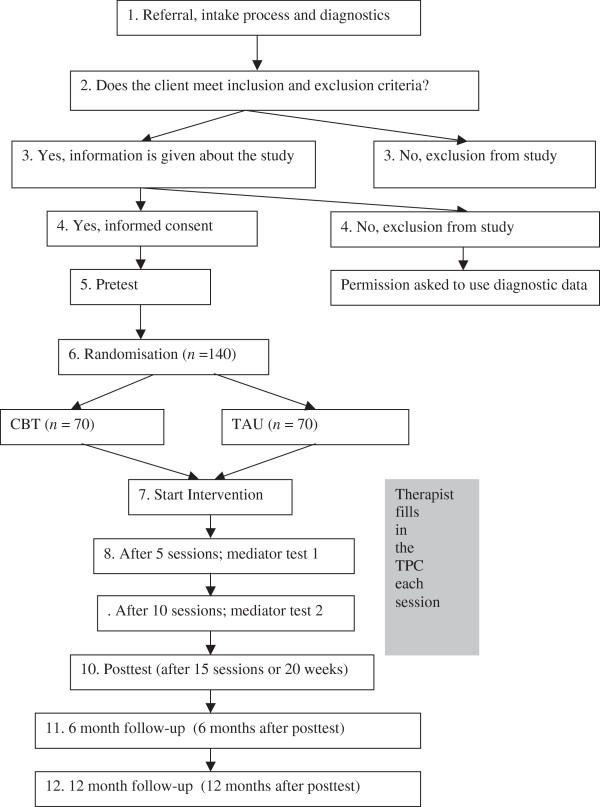
Participants’ flow through the study.

Adolescents who are diagnosed with a depression according to the K-SADS-PL [26,27], who meet inclusion criteria and do not meet the exclusion criteria are randomly assigned to either individual CBT or TAU. Random assignment per adolescent is executed using computer generated block randomisation and stratified per mental health centre. See Figure 2 for patient’s flow chart.

The design of this study is according to the guidelines specified by the Task force on promotion and dissemination (1995) and approved by the independent Medical Ethics Committee (METC) of the Utrecht Medical Centre at Utrecht University.

#### Sample size

Based on previous research [16] an effect size (Cohen’s d) of 0.53 is expected. Power calculations indicated that 70 adolescents per condition (assuming an alpha of 0.05, a statistical power, 1-beta, of 0.80 and a drop-out of 20%; power calculations in STATA) are required to detect a difference in depression diagnosis. In total 140 adolescents will be included.

#### Study sample

In total 140 referred clinically depressed adolescents aged 12 to 21 (*n* = 140) and their parents will be included in this study. If the adolescent is 18 years or older, parents will only be approached after the adolescent’s permission. The inclusion criteria for the depressive adolescents are: (1) a primary diagnoses of Depressive Disorder (regardless the severity: mild, moderate or severe) or Dysthymic disorder, (2) age 12 to 21 years, and (3) referred to one of the participating mental health institutions. The exclusion criteria are: (1) acute suicide risk, (2) drug abuse (as primary diagnosis), (3) pervasive developmental disorder (as primary diagnosis), (4) bipolar disorder (as primary diagnosis), (5) day care or admission to the clinical setting and (6) not fluent in Dutch, Turkish, Arabic or Berber language. If medication is used (for the depression or another disorder), the dosage should be kept constant during the intervention, unless medication is the control treatment.

Participants in both groups will be compared afterwards for their match on the following characteristics: age, ethnicity, gender, educational level parents and severity of the depression. If significant differences between two groups appear, these variables will be controlled for in analyses.

#### Recruitment

The participants are recruited from fourteen different specialized mental health care institutions spread all over the Netherlands. An experienced psychologist within the mental health centre informs the adolescents and their parents about the study. After written informed consent by the adolescent and his/her parents, a trained independent researcher carries out an interview to check for the inclusion and exclusion criteria.

#### Intervention

The experimental treatment is a protocolled individual CBT program, the D(o)epressie course. It consists of 15 weekly sessions of 45 minutes, two parent sessions after 3 and 9 weeks and a meeting with the parent at the end of treatment. The D(o)epressie course is, as the CWD-A, based on the social learning theory about the aetiology of depressions by Lewinsohn [28]. According to this theory, there is a connection between the number of positive interactions between a person and his environment on one hand, and depression on the other. A triggering event such as a stressful life event causes a negative spiral of less positive interactions leading to more negative thoughts and a deteriorating depressed mood. The aim of the intervention is to reduce depressive complaints in adolescents with a depressive disorder. Since depressive episodes are multi-factorial determined, the focus of the intervention is broad. The intervention contains representative components of CBT [29] namely: psycho-education (information about depression and the rationale for the aetiology of the complaints and the treatment of them), setting attainable goals (translate large goals into realistic short term goals), self monitoring (registration of the mood, activities and thoughts), activation (planning frequent, joyful activities), improving social skills and communication skills (improvement and stimulation of social behaviour), relaxation techniques, cognitive restructuring (identifying and changing unrealistic negative thoughts about the self, others and events), role play and problem solution skills (teaching the creation of solutions for problems via brainstorm, choosing, trying and evaluating) and relapse prevention. Exercises are executed within the sessions and are generalised into daily life by means of homework assignments. In the parent sessions, parents will receive psycho education and information on CBT. Therapists are not allowed to conduct treatments in both conditions.

The control treatment consists of TAU for clinical depression. After a short telephone survey we concluded that TAU in the Netherlands consists of: Interpersonal Therapy (IPT), family therapy, parent counselling, medication, mindfulness training, acceptance commitment therapy (ACT), psychodynamic therapy (short duration), (non-directive) counselling, creative therapy and running therapy, and CBT. However, in this study CBT is not allowed within TAU. The content of TAU and the treatment techniques used, are monitored.

#### Instruments

In Table 1, concepts, source and time of assessment of all used instruments are presented. The adolescent and parent complete the self-report questionnaires online at home. Both, the adolescent and the parent have a separate login code to secure privacy. The parents fill in a paper version of the cost diary at home. The therapist completes the questionnaires at the office.

**Table 1:** Instruments at different assessments and informants

	***Domain/concept***	***Instrument***	***Items***	***Source***			***Test***				
				***A.***	***P.***	***T.***	***Pre***	***Med***	***Post***	***Fu1***	***Fu2***
Primary outcome	Depression diagnosis	K-SADS		x	x		x		x	x	x
Secondary outcomes	Depression symptoms	CDI-II	28/17	x	x		x	x	x	x	x
	Depression severity	KSADS		x	x		x		x	x	x
		CGI-S	1			x	x	x	x		
	Depression Improvement	CGI-I	1			x	x	x	x		
	Global functioning	CGAS	1			x	x	x	x		
	Quality of life	EuroQol	6	x	x		x	x	x	x	x
	School or work	SQ	7	x			x		x	x	x
	Suicide Risk Taxation	SRT	6	x			x		x	x	x
	Comorbidity	K-SADS		x	x		x		x	x	x
		YSR	69	x			x		x	x	x
		CBCL	74		x		x		x	x	x
		SCARED	5	x	x		x		x	x	x
	Alcohol and drug use	AD	7	x			x		x	x	x
	Personality	Big 5	30	x			x		x		
	Life events	LES	23	x	x		x		x	x	x
	Parental depression	BDI-II	21		x		x		x	x	x
	Parental Psychopathology	ASR	69		x		x		x	x	x
		SCARED	4		x		x		x	x	x
	Parenting			x	x		x		x		
	-Responsivity	NOV	8								
	-Consistency	PDI	8								
	-Positive parenting	APQ	6								
	-Harsh discipline	SOG	8								
	-Psychological control	PCS	8								
	-Behavioural control	VTH	6								
	Competence	NOSI	5		x		x		x		
	Attachment	PARA	13	x	x		x		x		
	Conflict	NRI	6	x	x		x		x	x	x
Cost-effectiveness	Costs	Cost questionnaire			x		x		x	x	x
	Quality of life	EuroQol		x	x		x	x	x	x	x
Moderators	Demographic	Gender	8/12	x	x		x		x	x	x
	Characteristics adolescent	Age									
		Ethnicity									
		Education									
		Income									
	Depression severity	K-SADS		x	x		x		x	x	x
	Comorbidity	YSR			x		x		x	x	x
		CBCL		x			x		x	x	x
	Parental depression	BDI			x		x		x	x	x
	Parental Psychopathology	ASR			x		x		x	x	x
Mediators	Negative automatic thoughts	CNCEQ	16	x			x	x	x	x	x
	Cognitive Emotion regulation	CERQ	36	x			x	x	x	x	x
	Attribution style	CASQ	24	x			x	x	x	x	x
Treatment characteristics	Expectancy of treatment	PETS	7	x	x		x				
	Previous treatment	VEHI	8	x	x		x		x	x	x
	Satisfaction with treatment	SSS	4	x	x				x		
	Cooperation with treatment	CWT	5			x		x	x		
	Relationship with therapist	RWI	13	x				x	x		
	Treatment Integrity	observations							x		
	Therapy Procedures Checklist	TPC	51			x	Each session				
	Preferred treatment		1			x	x				

*Abbreviations*: *A* adolescent, *FU1* Follow-up1, *FU2* Follow-up2, *Med1* mediatortest1, *Med2* mediatortest2, *Post* posttest, *P* parents, *Pre* pretest, *T* therapist.

#### Primary outcome measures

The primary outcome measure is the presence of the depression diagnosis, as measured by the Kiddie-Schedule for Affective Disorders and Schizophrenia, present and lifetime version (K-SADS-PL) [26,27], a widely used semi-structured diagnostic interview. The K-SADS-PL assesses a wide range of diagnoses (present and life time) including their severity. The view of the adolescent, the parent and the independent clinician are taken into account. Concurrent validity of the K-SADS-PL is supported [26]. Also the interrater agreement is high (range 93% to 100%) and test –retest reliability is excellent for present and lifetime diagnoses of major depression (.77 to 100) [26]. Convergent validity of the depression screen criteria and the diagnoses generated with the K-SADS-PL was confirmed but divergent validity is only partly supported within an inpatient sample [30].

#### Secondary outcome measures

A broad range of secondary outcome measures will be assessed namely total symptoms of major depressive disorder or dysthymic disorder, severity and improvement of depression, global functioning, quality of life, suicide risk, comorbidity, alcohol and drug use, parental depression and psychopathology, parenting and conflicts. Costs, moderators, mediators and treatment characteristics will be assessed as well.

The degree of depressive symptoms are measured with a self report measure, the Child Depression Inventory-2 (CDI-2) [31,32]. The CDI-2 is a revision of the CDI [33,34] and was translated in Dutch. It was expanded with a version for the parents (CDI-P) [31] as well. The severity of the depression is rated by the independent clinician on the K-SADS-PL (see above) and by the therapist on the Clinical Global Impression-severity scale (CGI-S) [35]. Improvement of depression, in reference to the severity of the depression at the start of the treatment, is also rated by the therapist on the Clinical Global Improvement scale (CGI-I) [35]. Global functioning of the adolescent is measured by an independent clinician and therapist on the Children Global Assessment Scale (CGAS) [36,37]. The Dutch version of the EuroQol Questionaire (EQ-5D adolescent and parent version) [38] is used to establish quality of life as expressed in quality adjusted life years (QALYs). Apart from the K-SADS-PL, suicide risk is also assessed with a newly developed self-report questionnaire, which focuses on frequency of suicidal thoughts, wishes, plans and actions over the past two weeks.

Comorbidity and psychopathology is assessed with the K-SADS-PL, but also with the Youth Self Report scale (YSR) for adolescents and the Child Behavior Check List (CBCL) for parents [39,40]. Comorbidity between depression and anxiety is very high [12] therefore anxiety symptoms are also assessed separately with the Scared-5 [41].

Personality of the adolescent is assessed with the Quick Big Five Personality Inventory (QBF) [42]. For this study, we also constructed the Life Event Scale (LES) [43], which is a self-report measure about life events (including drug abuse, bereavement, maltreatment and suicide attempts), their date of occurrence and their impact on the adolescents well being.

Psychopathology of both parents is measured with the Adult Self-Report (ASR) [44]. The degree of depressive symptoms in parents is assessed with the Dutch version of the Beck Depression Inventory, second edition (BDI-II-NL) [45,46].

Parenting, in particular consistency, responsiveness, positive parenting, harsh discipline, psychological control and behavioral control is assessed with subscales of different instruments filled in by both the adolescent and the parent. The Parenting Dimensions Inventory (PDI) [47,48] was used to measure consistency, the degree to which the parent shows predictable discipline behaviour. The Nijmeegse Rearing Questionnaire (NOV) [49,50] measures responsiveness, the degree to which the parent is responsive for the needs, signals and condition of the child, and attachment, the degree to which the parent feels emotionally connected to the child. Positive parenting is measured with 6 items from the Alabama Parenting Questionnaire (APQ) [51]. The Ghent Parental Behavior Questionnaire (SOG) [52] assesses physical harsh discipline or physical punishment. Psychological control is assessed using the Psychological Control Scale (PCS) [53], which measures the degree in which the parent tries to control the child in an intrusive way. The degree in which parents monitor their children, that is behavioral control is measured with 6 items of the (Monitoring questionnaire, VTH) [54]. Competence, the parents’ perspective on their ability to address rearing practices, is established with the subscale competence of the Nijmeegse Parental Stress Index (NOSI) [55,56].

Attachment from child to parent is measured using 13 items of the Psychological Availability and Reliance on Adult (PARA) [57]. The degree of conflicts (quarrels, irritations and antagonism in the child–parent relationship) was measured with 6-item Network of relationship inventory (NRI) [58].

The economic evaluation is done by registration of costs in a cost diary based on the Trimbos Institute and Institute of Medical Technology Assessment Questionnaire on Costs Associated with Psychiatric Illness (TiC-P) [59] and PRODISQ [60]. The registered costs are directly related to health care or indirect health care (out-of-pocket costs, costs of informal care) and direct costs outside health care (monetary value of production losses caused by absence and reduced productivity). The costs will be considered separately from the perspective of mental health and from society. The mental health costs are the costs, which are credited to the mental health care budget, the decision-maker’s perspective. The societal costs are the costs of direct (mental) health care as well as indirect costs such as lost productivity, school absent and out-of-pocket costs.

Potential moderators that are analysed are severity of the depression, comorbidity, parental depression and psychopathology and demographic variables. Demographic information is gathered by adding questions about gender, age, ethnicity, education level and family income to the self-report questionnaires.

Three mediators are investigated namely negative automatic thoughts (CNCEQ) [61], cognitive emotion regulation (CERQ) [62] and attribution style (CASQ) [63]. The Cognitive Negative Cognitive Error Questionnaire (CNCEQ) measures cognitive errors namely the underestimation of the ability to cope, personalizing without mind reading, selective abstraction, over generalizing and mind reading. The Cognitive Emotion Regulation Questionnaire (CERQ) measures a broad set of cognitive emotion regulation strategies which are used in response to the experience of threatening or stressful life events; Self-blame, Other-blame, Rumination, Catastrophizing, Positive refocusing, Planning, Positive reappraisal, Putting into perspective and Acceptance. The Children’s Attributional Style Questionnaire (CASQ) is a self-report measure with three dimensions of attribution; internal- external, stable-unstable and global- specific.

Several non-specific treatment variables will be investigated. The client’s credibility, expectancy and involvement regarding treatment are assessed with the Parent Expectancies for Therapy Scale (PETS) [64] which was revised for adolescents. Previous treatments for depression, including complementary and self-help treatments, are administered with the inventory of History of Treatments (VEHI) [65]. Satisfaction with treatment is measured with the Service Satisfaction Scale (SSS) [66]. The Cooperation With Treatment scale (CWT) [67] is used to assess the degree of cooperation with treatment as observed by the therapist. The quality of the therapeutic alliance is assessed with the Therapy Alliance Scale for Adolescents (TASC) [68]. The content of treatment is assessed in both conditions with the Therapy Procedure Checklist (TPC) [69]. Treatment integrity will be established by recording two randomly chosen sessions that are observed and rated.

### Statistical analyses

Missing values will be imputed. Intent-to-treat as well as completer analyses will be conducted. The effect of the intervention is analyzed with a chi-square test on the dichotomous primary outcome measure, presence of the depression diagnosis (K-SADS-PL). The cost-effectiveness analyses will be based on the comparison of costs and effects in both conditions and will be done according the international guidelines [70]. The cost- effectiveness analyses will be done separately from the perspective of mental health and society over a period of 6 months and 1 year. The effects of societal costs will be expressed in years to live, corrected for quality of life (QALYs). The costs of CBT versus CAU will be expressed in 1) incremental costs per QALY and 2) incremental costs per adolescent with a depression in full remission.

The secondary continuous outcome measures will be analyzed with repeated measures MANCOVA using the pre-test as a covariate as is recommended for RCT’s with pre-, post- and follow-up measurements [71]. For each questionnaire, the effect size (Cohen’s d) from pre- to post-treatment is defined as (M_pre_-M_post_)/SD_pooled_, where SD_pooled_ = [(SD_pre_^2^ + SD_post_^2^)/2]. CBT versus TAU pre-post effect sizes are calculated as follows [(M_CBT post_-M_TAU post_)/SD_pooled post_] – [ (M_CBT pre_-M_TAU pre_)/SD_pooled pre_]. Furthermore, analyzes will be conducted to establish clinical significance and the reliable change index [72].

Moderators will be analysed by multi-group analyses for dichotomous variables. The continuous variables will be analysed using hierarchic regression analyses. Mediator effects will be analysed using hierarchical regression analyses and structural equation modelling.

## Discussion

As Weisz [13] pointed out, it is not enough to replicate studies to increase our knowledge of effective treatment of adolescent depression. It is necessary to be innovative and to go further and “pushing the boundaries of what has been done” [13]. In this study the design is innovative in several regards.

First, the target population consists of referred clinically depressed adolescents and the treatment is conducted within routine mental health care services with routine care professionals, in the Netherlands. Therefore, CBT is investigated under real life conditions.

Second, the control condition is an active condition, namely treatment as usual (TAU) within routine clinical care, not a waiting list condition or just one specific treatment such as medication. TAU being the control condition is a more rigorous test of effectiveness of CBT. As a secondary spin off, the collected data will also enable a detailed description of TAU without CBT.

Third, long term effects of CBT versus TAU will be examined, up to one year post treatment. This is important because of possible sleeper effects [73] and recurrence of depressive symptoms.

Fourth, cost-effectiveness was investigated for a group CBT program “Coping with Stress”, but only in adolescents with a subclinical depression and as a prevention program [24]. Cost-effectiveness of CBT versus TAU in adolescents with clinical depression to our knowledge is not yet investigated. Also productivity losses have never been taken into account. The cost-effectiveness is of increased importance because of diminishing budgets in mental health care. Perhaps even more important, in this study the costs of clinical depression in adolescents are determined.

Fifth, although it is very important to know why a treatment works and for whom it works, few studies actually addressed this topic. In this study potential mediators and moderators will be investigated.

As a result of the design, we anticipate several problems, which may become limitations of this study if not properly addressed. To ensure the quality of the study, these problems and their solutions, which are undertaken to minimize the limitations, are discussed.

First, content of TAU and the techniques used are difficult to monitor. Assessment of the therapeutic procedures used during each session by the therapist, provides detailed information on the components of the treatment. Also two sessions are recorded at random, observed and rated in detail to assess content of treatment and techniques used.

Second, comparing CBT to an active control condition may lead to a lower mean effect size than CBT versus a non-active control condition [13]. The TAU condition in this study is heterogeneous and it contains also evidence-based treatments such as Interpersonal therapy. As a result the ES can be even smaller, so we increased the power accordingly.

Third, to include 140 adolescents within 18 months, cooperation with a large group of mental health institutions is necessary. Furthermore, we try to minimize the effort the adolescent and their parents have to put into the study by using online assessments of the self-report questionnaires, while ensuring secrecy. Since the K-SADS-PL is assessed in vivo, not only self-report, but also observations by an independent clinician are taken into account.

Fourth, non-response may be a huge problem because of the multiple assessments and informants. Online assessment enables the researchers to monitor the progress per assessment per informant. They can react immediately to non-response and increase the response rate accordingly. This transparent logistic process of data collection, data transportation and data file construction reduces the risk of mistakes, missing data and contributes to the quality of the data [74].

Fifth, as it is not allowed to conduct CBT within the TAU condition it is inevitable that TAU is changed in this regard. This is a methodological problem, which may lead to bias, but cannot be solved properly. Although we are fully aware of this methodological problem we accept it because of the advantages of an RCT in a naturalistic setting. The preferred choice of treatment by the multidisciplinary team for every included depressed adolescent is registered and enables us to take into account the preferred choice of treatment.

Sixth, the total amount of face-to-face contact within TAU can be different from CBT. Also the time period in which the treatment is completed can differ within TAU and CBT. The amount of face-to-face contact in minutes and the time period of the treatment will be taken into account as potential moderating factors.

## Competing interests

The authors declare that they have no competing interests.

## Authors’ contributions

DB, YS and ALvB obtained funding for the study. All authors (DB, YS, MD and ALvB) contributed to the design of the study. DB and YS coordinate the recruitment of participants and data collection during the study. YS made the adaptation of the CBT treatment and supervises the CBT treatment. YS wrote the manuscript on basis of the initial research template written by DB and YS. All authors contributed to the writing of the manuscript. All authors read and approved the final manuscript.

## Pre-publication history

The pre-publication history for this paper can be accessed here:

http://www.biomedcentral.com/1471-244X/13/314/prepub
